# 2D MOF Nanoflake-Assembled Spherical Microstructures for Enhanced Supercapacitor and Electrocatalysis Performances

**DOI:** 10.1007/s40820-017-0144-6

**Published:** 2017-03-28

**Authors:** Huicong Xia, Jianan Zhang, Zhao Yang, Shiyu Guo, Shihui Guo, Qun Xu

**Affiliations:** 1 College of Materials Science and Engineering, Zhengzhou University, Zhengzhou, 450001 People’s Republic of China; 2 Key Laboratory of Advanced Energy Materials Chemistry (Ministry of Education), Nankai University, Tianjin, 30071 People’s Republic of China; 3 State Key Laboratory of Inorganic Synthesis and Preparative Chemistry, Jilin University, Changchun, 130012 People’s Republic of China

**Keywords:** Metal–organic frameworks, Nanoflakes, Spherical microstructure, Supercapacitor, Oxygen reduction reaction

## Abstract

Metal–organic frameworks (MOFs) are of great interest as potential electrochemically active materials. However, few studies have been conducted into understanding whether control of the shape and components of MOFs can optimize their electrochemical performances due to the rational realization of their shapes. Component control of MOFs remains a significant challenge. Herein, we demonstrate a solvothermal method to realize nanostructure engineering of 2D nanoflake MOFs. The hollow structures with Ni/Co- and Ni-MOF (denoted as Ni/Co-MOF nanoflakes and Ni-MOF nanoflakes) were assembled for their electrochemical performance optimizations in supercapacitors and in the oxygen reduction reaction (ORR). As a result, the Ni/Co-MOF nanoflakes exhibited remarkably enhanced performance with a specific capacitance of 530.4 F g^−1^ at 0.5 A g^−1^ in 1 M LiOH aqueous solution, much higher than that of Ni-MOF (306.8 F g^−1^) and ZIF-67 (168.3 F g^−1^), a good rate capability, and a robust cycling performance with no capacity fading after 2000 cycles. Ni/Co-MOF nanoflakes also showed improved electrocatalytic performance for the ORR compared to Ni-MOF and ZIF-67. The present work highlights the significant role of tuning 2D nanoflake ensembles of Ni/Co-MOF in accelerating electron and charge transportation for optimizing energy storage and conversion devices.

**Electronic supplementary material:**

The online version of this article (doi:10.1007/s40820-017-0144-6) contains supplementary material, which is available to authorized users.

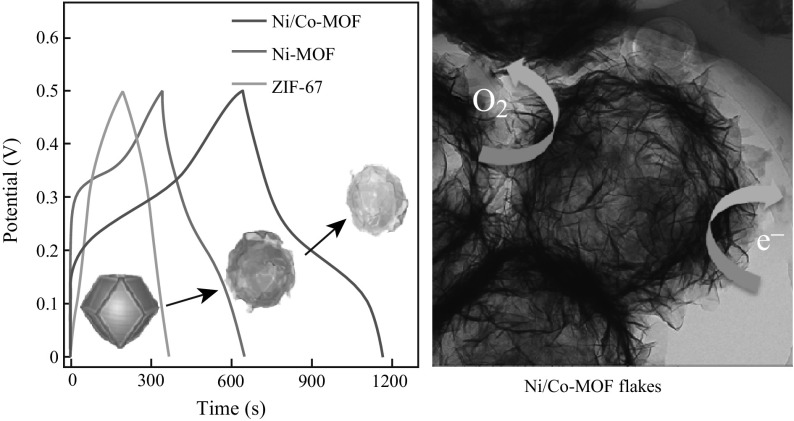

## Highlights

- A solvothermal method was used to improve the conductivity and electrochemical activity of metal–organic framework (MOF) materials by tuning their morphology and components.
- Ni/Co-MOF nanoflakes exhibit remarkably enhanced performances including enhanced electrocatalytic performance for the oxygen reduction reaction.
- The synthetic strategy driven by rational design gives the first example of exploring MOF-derived nanomaterials to achieve improved efficiency energy storage and conversion devices.

## Introduction

The design and building of metal–organic frameworks (MOFs) with controllable structures have received significant attention due to their wide range of applications such as in gas storage and separation [1–4], optoelectronics and energy storage [5–10], catalysis [11–17], and drug delivery and imaging [18–20]. With their exceptionally large surface area, abundant micropores, and variable sites for redox reactions, MOFs are considered advanced promising electrode materials for electrochemical energy storage and conversion devices such as batteries, supercapacitors, and fuel cells [8, 21–27]. The biggest problem facing individual MOFs is that they suffer from relatively low conductivity and poor electrolyte ion transport behavior, thereby restricting their efficiency for energy storage and conversion. A general approach to addressing this issue is to apply MOFs as sacrificial templates to generate porous carbon with metal or metal oxides, which can afford high conductivity and electrochemical reactivity. While high-temperature treatment inevitably results in cost increase, the intricate porous structure of MOFs cannot be employed generally [22, 28–31]. The direct application of a series of MOFs as electrode materials for supercapacitors, presented in Yaghi and co-workers’ pioneering study, seems promising [32], but sluggish kinetics and poor long-term stability for electrochemical capacitors and electrochemical catalysis greatly limit their utilization.

Recent studies revealed that two-dimensional (2D) nanomaterials with short pathways for mass transport and multiple metallic ions create an opportunity for energy storage and conversion devices relative to their counterparts with other dimensionalities. The electron confinement in two dimensions of the ultrathin 2D nanomaterials renders compelling electronic properties [33–36]. Prominent examples include the MOF@graphene oxide designed for the lithium-sulfur battery that functioned as a battery separator to selectively sieve Li^+^ ions while blocking polysulfides [9], the 2D porphyrin paddlewheel framework-3 (PPF-3) nanoflake successfully used as an electrode for a supercapacitor [37], the 2D metal oxide/hydroxide graphene nanohybrids that exhibited outstanding catalytic behavior for the oxygen reduction reaction (ORR) [38], and metal-nitrogen-containing mesoporous carbon/graphene nanoflakes exhibiting enhanced ORR performance [39]. Despite the fact that these state-of-the-art 2D nanoarchitectures show great potential in optimizing energy storage and catalysis, they usually show limited performance because of the following two issues: (I) most MOFs possess micropores with diameters less than 2 nm, thus blocking the transport of atoms, ions, and large molecules. Therefore, the coexistence of micropore–mesopore–macropores in MOFs is highly desired; and (II) the reactivity of MOFs on the pseudocapacitance and catalytic performance should be enhanced by optimizing the valence variability of the metallic ions as redox centers. In this regard, it will be of great significance to develop MOFs with multiple structures that consist of hierarchical pores and 2D nanoflakes, and also a 3D large interconnected network that can afford efficient charge, mass exchange, and low internal resistance.

Herein, we report a facile strategy to synthesize 2D MOF nanoflake-assembled spherical microstructures composed of ultrathin Ni/Co- and Ni-imidazolate framework nanoflakes as subunits (simplified as Ni/Co-MOFs nanoflakes). This unique superstructure with 3D accessible sites, maximized surface area, and synergistic effect of dual metallic (Ni^3+^/Ni^2+^ and Co^3+^/Co^2+^) ions is highly beneficial in enhancing electrochemical storage and conversion. As a result, the Ni/Co-MOFs nanoflakes exhibit remarkable performances with a specific capacitance of 530.4 F g^−1^ at 0.5 A g^−1^ in 1 M LiOH aqueous solution, 1.72 and 3.15 times higher than that of Ni-MOF nanoflakes (306.8 F g^−1^) and ZIF-67 (168.3 F g^−1^), respectively; good rate capability and robust cycling performance with no capacity fading after 2000 cycles. Additionally, Ni/Co-MOF nanoflakes show higher electrocatalytic activity for the ORR than Ni-MOF nanoflakes and ZIF-67. The smart synthetic strategy employing rational design gives the first example of exploring MOF-derived nanomaterials in achieving more efficient energy storage and conversion devices.

## Materials and Methods

### Chemicals

Cobalt nitrate hexahydrate (Co(NO_3_)_2_·6H_2_O) was purchased from Zhengzhou Chemical Reagents Co., Ltd. Anhydrous methanol was purchased from Tianjin Chemical Reagents Co., Ltd. 2-Methylimidazole (MW = 82.10, C_4_H_6_N_2_) was purchased from Sigma-Aldrich. Nickel(II) nitrate hexahydrate (Ni(NO_3_)_2_·6H_2_O) was purchased from Sinopharm Chemical Reagent Co., Ltd. All chemicals used were of analytical grade and used without further purification.

### Synthesis of ZIF-67

ZiF-67 was synthesized according to the previous literature [40]. A solution of 2-methylimidazole (7.5 mM, 15 mL) in methanol was slowly added to 15 mL of a Co(NO_3_)_2_·6H_2_O (1.9 mM) methanol solution using a syringe at room temperature. After ultrasonic irradiation for 15 min, the ZIF-67 nanocrystals were separated via centrifugation.

### Synthesis of Ni-MOF Nanoflakes

Ni-MOF nanoflakes were synthesized by a solvothermal method. The as-prepared ZiF-67 nanocrystals were dispersed in 15 mL methanol followed by the addition of 15 mL Ni(NO_3_)_2_·6H_2_O methanol solution (1.9 mM). The above mixture was transferred to a Teflon-lined stainless-steel autoclave and kept at 120 °C for 1 h. Finally, the product was obtained by centrifugation, washed three times with methanol, and dried at 60 °C for 12 h.

### Synthesis of Ni/Co-MOF Nanoflakes

As-prepared ZiF-67 nanocrystals were dispersed in 15 mL methanol followed by the addition of 15 mL methanol containing Ni (NO_3_)_2_·6H_2_O (0.95 mM) and Co(NO_3_)_2_·6H_2_O (0.95 mM). The product was obtained by centrifugation, washed three times with methanol, and dried at 60 °C for 12 h.

### Characterization

The morphologies of the samples were studied by field-emission scanning electron microscopy (FE-SEM, JEORJSM-6700F) and transmission electron microscopy (TEM, FEI Tecnai G2 20) with an accelerating voltage of 200 kV. Powder XRD patterns were collected using a Y-2000 X-ray diffractometer with copper *K*_α_ radiation (*λ* = 1.5406 Å) at 40 kV and 40 mA. Fourier transform infrared (FTIR) spectra of the products were recorded on a TENSOR 27 FTIR spectrometer (Bruker) in the absorption mode with a resolution of 2 cm^−1^. The X-ray photoelectron spectroscopy (XPS) measurements were performed with an ESCA LAB 250 spectrometer using a focused monochromatic Al-*K*_α_ line (1486.6 eV) X-ray beam with a diameter of 200 μm. Thermal gravimetric analysis (TGA) was conducted on an SMP/PF7548/MET/600 W instrument from 50 to 800 °C with a heating rate of 10 °C min^−1^ in a nitrogen atmosphere.

### Electrochemical Measurements

#### Supercapacitor Measurements (Three-Electrode System)

The capacitance performances of the samples were evaluated with a three-electrode system on an electrochemical workstation (CHI 760E, CH Instrument, China) at room temperature. The suspension with active materials at a concentration of 1.0 mg mL^−1^ was prepared by sonicating 1 mg of active materials in 1 mL ethanol containing Nafion (Sigma-Aldrich, 5 wt%) at a volume ratio of 995:5. Then, 20 μL of ink was dropped onto a glassy carbon disk (diameter 5 mm) and dried thoroughly in air, resulting in a catalyst loading of 0.1 mg cm^−2^. The auxiliary and reference electrodes were Pt wire and Ag/AgCl, respectively. The electrochemical measurements were carried out in 1 M LiOH solution.

The potential range for cyclic voltammetry (CV) tests was 0–0.5 V, and the scan rates were 5, 10, 25, 50, 75, 100, 150, and 200 mV s^−1^. Galvanostatic charge–discharge (GCD) measurements were done from 0 to 0.5 V at different current densities of 0.5, 1, 2, 4, 6, 8, and 10 A g^−1^. The gravimetric specific capacitance *C* (F g^−1^) based on the discharge curves was calculated by:

1 $$C = \left( {I \times \Delta t} \right)/\left( {\Delta V \times m} \right)$$

where *I* is the discharge current (A), Δ*t* is the discharge time (s), Δ*V* is the potential window (V), and *m* is the mass of active materials on the test electrode (g).

#### Oxygen Reduction Reaction (ORR)

To prepare the working electrode, 5 mg of catalyst and 5 mg carbon black (Alfa Aesar, 99.9+ wt%) were dispersed in a mixture of 950 μL ethanol and 50 μL Nafion (Sigma-Aldrich, 5 wt%) under sonication for 30 min to obtain a homogeneous slurry. Then, 8 μL of this catalyst ink was loaded onto a glassy carbon rotating disk electrode of diameter 5 mm, resulting in the catalyst loading of 0.2 mg cm^−2^. The electrode was dried under dissolvent conditions for 5 h.

Electrochemical impedance spectral measurements were carried out in the frequency range from 100 kHz to 10 mHz on a CHI 760E electrochemical workstation. Cyclic voltammetry (CV) and rotating disk electrode (RDE) measurements (Pine Research Instruments, USA) were conducted using a standard three-electrode system. The catalyst-coated glassy carbon electrode, an Ag/AgCl electrode in saturated KCl solution, and Pt wire were used as the working, reference, and counter electrodes, respectively. The electrolyte was 0.1 M potassium hydroxide (KOH) aqueous solution. The potential measured against the Ag/AgCl electrode was converted to the potential versus the reversible hydrogen electrode (RHE) according to *E* (vs. RHE) = *E* (vs. Ag/AgCl) + 0.197 + 0.059 pH. All measurements were carried out at room temperature.

For the ORR at an RDE, the working electrode was scanned cathodically at a rate of 10 mV s^−1^ at different rotating speeds from 400 to 2500 rpm in O_2_-saturated 0.1 M KOH aqueous solution. Koutecky–Levich (K–L) plots were analyzed at various electrode potentials. The slopes of their linear fit lines were used to calculate the electron transfer number (n) on the basis of the K–L equation:

2 $$\frac{1}{J} = \frac{1}{{J_{\text{L}} }} + \frac{1}{{J_{\text{K}} }} = \frac{1}{{B\omega^{0.5} }} + \frac{1}{{J_{\text{K}} }}$$

3 $$J_{\text{L}} = 0.62nFC_{0} D_{0}^{2/3} \omega^{0.5} \nu^{ - 1/6}$$

4 $$B = 0.62nFC_{0} D_{0}^{2/3} \nu^{ - 1/6}$$

5 $$J_{\text{K}} = nFkC_{0}$$

where *J* is the measured current density, *J*_L_ and *J*_K_ are the diffusion- and kinetic-limited current densities, *ω* is the rotation speed (rad s^−1^), *n* is the transferred electron number, *F* is the Faraday constant (96,485 C mol^−1^), *C*_0_ is the O_2_ concentration in the electrolyte (1.26 × 10^−6^ mol cm^−3^), *D*_0_ is the diffusion coefficient of O_2_ in the electrolyte (1.93 × 10^−5^ cm^2^ s^−1^), and *v* is the kinetic viscosity (0.01009 cm^2^ s^−1^).

## Results and Discussion

**Scheme 1:**
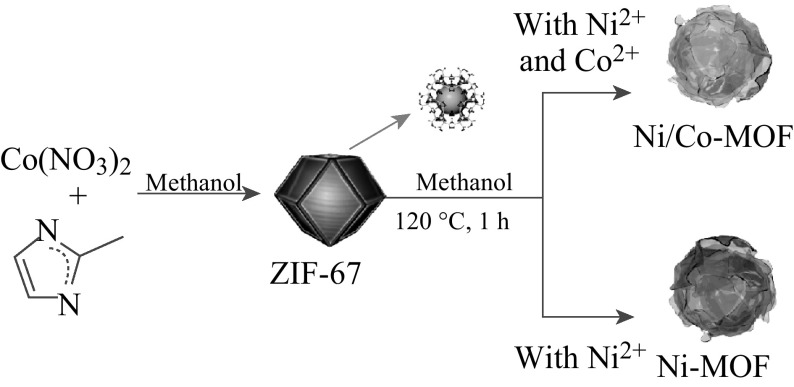
Schematic illustration of the synthesis of Ni/Co-MOF nanoflakes and Ni-MOF nanoflakes

Scheme 1 illustrates the wet-chemical protocol for synthesizing the 2D Ni/Co-MOF nanoflake-assembled superstructure via morphology transformation of ZIF-67([Co(MeIm)_2_]_n_) (MeIm = methylimidazole). Typically, ZIF-67 rhombododecahedron nanoseeds with sizes of around 400 nm were prepared employing Co(NO_3_)_2_ as metallic source, 2-methylimidazole as organic linker, and methanol as solvent (Fig. S1a). Sequentially, ZIF-67 rhombododecahedron nanoseeds were dispersed in methanol followed by the addition of solutions of Ni(NO_3_)_2_ and Co(NO_3_)_2_ in methanol. The parent ZIF-67 would readily evolve into a distinctive hollow nanocage after 60 min of solvothermal treatment in methanol. In the initial stage, we suggest that Ni^2+^ partly substituted Co^2+^ in the framework, which could retain the ZIF-67 crystalline lattice. Over time, the ZIF-67 rhombododecahedron were gradually etched and the Ni/Co-MOF nanoflakes were simultaneously formed and covered the surface of the polyhedron, which was accompanied by a size increase in the inner core from 400 to 500–700 nm. It was observed that all of the solid ZIF-67 had transformed to 2D hollow MOF nanoflake spherical microstructures.

**Fig. 1:**
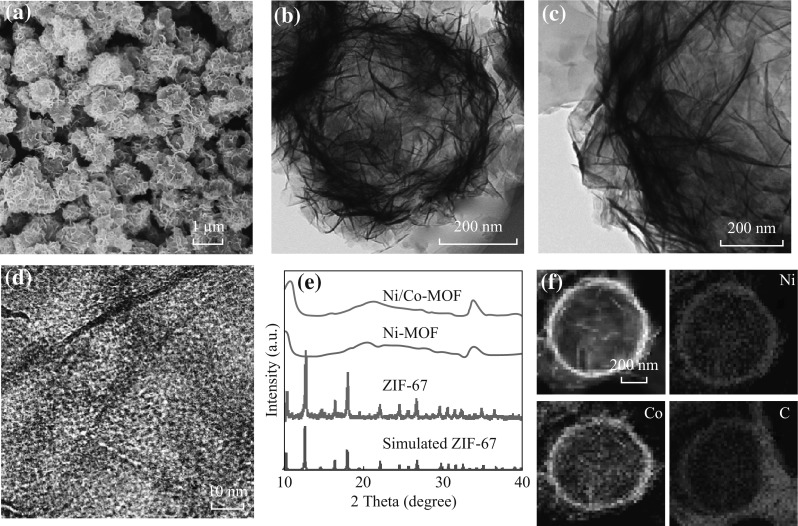
**a** SEM images of Ni/Co-MOF nanoflakes. **b**, **c** TEM images of Ni/Co-MOF nanoflakes. **d** Large-magnified TEM and SAED patterns of Ni/Co-MOF nanoflakes. **e** XRD patterns of Ni/Co-MOF nanoflakes, Ni-MOF nanoflakes, and ZIF-67. **f** STEM-energy-dispersive spectroscopy (EDS) mapping of Ni/Co-MOF nanoflakes

**Fig. 2:**
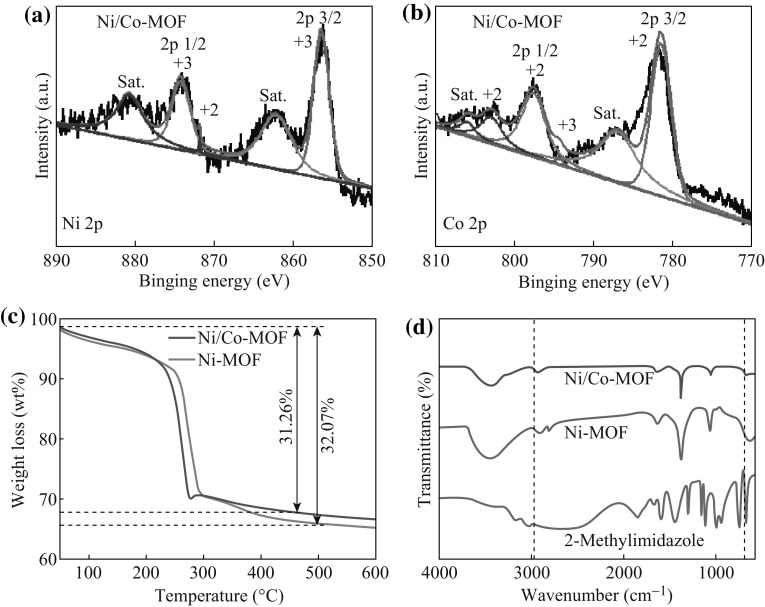
**a** Ni 2*p* XPS spectra and **b** Co 2*p* spectra of Ni/Co-MOF nanoflakes. **c** TGA of Ni/Co-MOF nanoflakes and Ni-MOF nanoflakes. **d** FTIR spectra of Ni/Co-MOF nanoflakes, Ni-MOF nanoflakes, and 2-methylimidazole

As demonstrated in the TEM (Fig. S1b, c) and SEM (Fig. 1a) images, both Ni/Co-MOF nanoflakes and Ni-MOF nanoflakes show distinctive hollow nanocages assembled by overlapping nanoflakes and hollow superstructures are well-defined with the average diameter of 500–700 nm corresponding to the ZIF-67 precursor (~400 nm). Taking a closer look at the detailed structure, each spherical microstructure is essentially a 3D structure comprised of numerous building blocks of 2D ultrathin nanoflakes (Fig. 1b, c). To further figure out the detailed structure of the Ni/Co-MOF nanoflakes, magnified TEM images and XRD patterns of Ni/Co-MOF nanoflakes were collected (Fig. 1d, e). It was found that an individual nanosheet of Ni/Co-MOF nanoflakes consists of numerous white lines with widths of <1 nm implying the microporous structure of the Ni/Co-MOF nanosheets, which greatly benefits mass transport during electrochemical reactions. The XRD patterns (Fig. 1e) of the Ni/Co-MOF nanoflakes, Ni-MOF nanoflakes, and ZIF-67 were measured. The peaks of the parent ZIF-67 seeds matched the patterns of simulated ZIF-67, whereas the characteristic peaks of ZIF-67 (12.76° and 18.11°) disappeared and two broad peaks emerged at 10.48° and 33.96° when Ni/Co-MOF nanoflakes and Ni-MOF nanoflakes formed. It was proposed that the coordination mode between Ni^2+^ and Co^2+^ ions and 2-methylimidazole was influenced by the involvement of methanol molecules in the unit cell [41]. Such influences result in structural evolution from a rhombododecahedron to the 2D MOF nanoflake-assembled spherical microstructure. The investigation of the valence states of Ni and Co was carried out using XPS (Fig. 2a, b, S2). The characteristic bands at 873.3 and 796.9 eV are the fingerprints of Ni^2+^ and Co^2+^ in tetrahedral coordination, implying the persistence of the coordination mode during the phase transformation [42–45]. The Ni 2*p* spectrum of the Ni/Co-MOF can be deconvolved into two spin–orbit doublets. The first doublet at 856.6 eV and the second at 874.3 eV could be assigned to Ni^3+^ [46]. The Ni^3+^/Ni^2+^ ratio is ~1.22 for Ni/Co-MOF. The binding energy at 781.6 eV corresponds to the spin–orbit characteristic of Co^2+^. The spectral Co^3+^/Co^2+^ ratio obtained from their respective main lines is ∼0.78 for the Ni/Co-MOF. The elemental distributions of Ni/Co-MOF and Ni-MOF nanoflakes were further confirmed from STEM-energy-dispersive spectroscopy (EDS) mapping (Fig. S2). As shown in Fig. 1f and Fig. S2, Co, Ni, C, and N were uniformly distributed on the overall nanoflakes, and the Ni and Co elements were in the loaded Ni/Co-MOF nanoflakes.

The TGA data of Ni/Co-MOF and Ni-MOF nanoflakes were collected under air at the heating rate of 10 °C min^−1^ from room temperature to 800 °C. The slight weight loss (<8 wt%) before 220 °C is attributed to water that is bonded to the imidazole group of the MOF framework via H-bonds. The typical weight loss of Ni/Co-MOF and Ni-MOF nanoflakes calculated from the TGA data (Fig. 2c) was determined to be 31.26 and 32.07 wt% at 250 and 220 °C, respectively, attributed to the removal of the organic ligands. FTIR spectra of Ni/Co-MOF and Ni-MOF nanoflakes were measured to identify the surface functional groups and observe the formation of coordinated polymers. As shown in Fig. 2d, the FTIR spectrum has been studied extensively and every absorption peak was assigned to its corresponding vibration. The characterization peaks at 2923 and 584 cm^−1^ were attributed to the aliphatic C–H stretch and the C=N stretching vibrations of 2-methylimidazole. Therefore, the stretching of aliphatic C–H and vibration of C=N in 2-methylimidazole at 624 and 3100 cm^−1^ shifted to 584 and 2923 cm^−1^ after assembling Ni/Co-MOF or Ni-MOF, indicating the strong interaction of C=N and C–H groups in 2-methylimidazole with the Ni^2+^ or Co^2+^ ions [47]. Based on the above analysis, the linker between the Ni^2+^ and Co^2+^ and 2-methylimidazole remained unchanged during phase transformation.

**Fig. 3:**
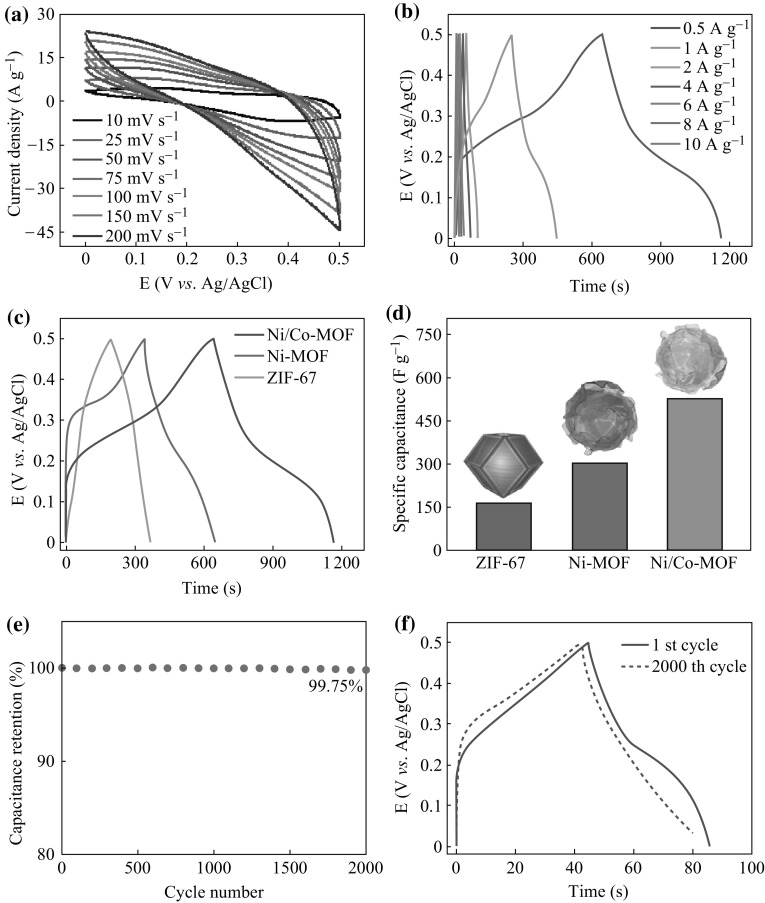
**a** Cyclic voltammograms of as-prepared Ni/Co-MOF nanoflake electrodes at different scan rates in 1 M LiOH solution. **b** Galvanostatic charge–discharge curves of Ni/Co-MOF nanoflakes at various currents in 1 M LiOH solution. **c** Galvanostatic charge–discharge curves comparison of Ni/Co-MOF nanoflakes, ZIF-67, and Ni/Co-MOF nanoflakes at various current densities in 1 M LiOH solution. **d** Specific capacitances of Ni/Co-MOF nanoflakes, Ni-MOF nanoflakes, and ZIF-67 electrodes at 0.5 A g^−1^. **e** Cycling performance of Ni/Co-MOF nanoflakes at a current density of 2 A g^−1^. **f** Galvanostatic charge–discharge curves of Ni/Co-MOF nanoflake supercapacitor before and after 2000 cycles measured at 2 A g^−1^

The unique structure of the Ni/Co-MOF nanoflakes and Ni-MOF nanoflakes inspired us to evaluate their electrochemical performance for a supercapacitor with a three-electrode system in 1 M LiOH aqueous electrolyte. As shown in Fig. 3a and Fig. S3, CV curves of Ni/Co-MOF nanoflakes present roughly rectangular shapes and reversible bumps, suggesting the combination of electric double-layer capacitance (EDLC) and a pseudocapacitive reaction. It should be noted that the roughly rectangular shape of the Ni/Co-MOF nanoflakes was maintained even at a high potential scan rate of 200 mV s^−1^, indicating pure EDLC behavior and rapid formation of the double layer even at high rates. The appearance of nearly symmetric galvanostatic charge–discharge curves in Fig. 3b indicates that the unique electrode has a low polarization and high charge–discharge columbic efficiency. Significantly, Fig. 3c, d shows that the Ni/Co-MOF nanoflakes exhibit a high specific capacitance of 530.4 F g^−1^ at 0.5 A g^−1^, 3.15 and 1.72 times higher than those of the Ni-MOF nanoflakes (306.8 F g^−1^) and ZIF-67 (168.3 F g^−1^). As shown in Table S1, the Ni/Co-MOF nanoflakes showed a much higher specific capacitance than many previously reported MOF-based materials such as worm-like Co-MOF [23], curled sheet-like N-doped Zn-MOF [48], sodalite-structured ZIF-67 [49], and honeycomb-like Co-MOF [50]. Figure 3e, f presents the cycling performance of the Ni/Co-MOF nanoflakes at a current density of 2 A g^−1^. The Ni/Co-MOF nanoflakes show a very stable capacitance (99.75% of the original capacitance) after 2000 cycles of charging and discharging, indicating long-term electrochemical stability. This is further confirmed by the inconspicuous change between the charging–discharging curves of the first and 2000th cycles. Considering their high specific capacitance and robust electrochemical stability, the Ni/Co-MOF nanoflakes present promise as an advanced electrode candidate for supercapacitors. Obviously, the Ni/Co-MOF and Ni-MOF nanoflakes exhibit better supercapacitor performance than ZIF-67, revealing the vital role of the micropore–macropore coexisting in 2D-3D MOF nanoflakes for enhancing the energy storage. According to the property of the CV and GCD curves of Ni/Co-MOF (potential window, oxide reduction peak, and pseudocapacitive behavior), possible active sites are the Co^3+^/Co^2+^ and Ni^3+^/Ni^2+^ ions in the structure. As shown in Fig. S4, the Ni/Co-MOF nanoflakes show terrible capacitance (70.85% of the original capacitance) after 3000 cycles of charging and discharging, which is probably due to destruction of the morphology.

**Fig. 4:**
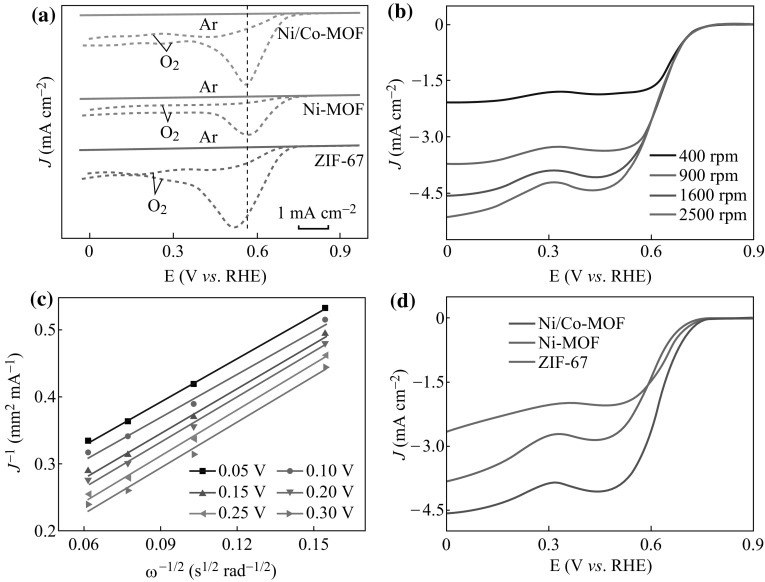
**a** CV curves in Ar-saturated (*solid curves*) and O_2_-saturated (*dashed curves*) solutions with a sweep rate of 50 mV s^−1^. **b** RDE polarization curves of Ni/Co-MOF nanoflakes at different rotation speeds. Scan rate: 10 mV s^−1^. **c** Koutecky–Levich plots of Ni/Co-MOF nanoflakes at various potentials. **d** RDE polarization curves of samples at a rotation rate of 1600 rpm. Scan rate: 10 mV s^−1^

The as-prepared Ni/Co-MOF nanoflakes also work well as advanced non-metal catalysts for enhancing the ORR. Figure 4a shows the CVs of Ni/Co-MOF nanoflakes, Ni-MOF nanoflakes, and ZIF-67 performed in Ar- and O_2_-saturated 0.1 M KOH solutions. The auxiliary and reference electrodes were Pt wire and Ag/AgCl, respectively. Compared with the featureless character in the Ar-saturated solutions, catalytic peaks were found in the O_2_-saturated solutions. A well-defined oxygen reduction cathodic peak at about 0.57 V can be clearly observed for the Ni/Co-MOF nanoflakes in the O_2_-saturated solution, which is positively shifted about 20 and 50 mV compared to the Ni-MOF nanoflakes (0.55 V) and ZIF-67 (0.52 V), respectively, indicating the best effective electrochemical reduction of oxygen for the Ni/Co-MOF nanoflakes. As shown in Fig. 4b, to gain the dynamic information about the ORR, the electrocatalytic abilities were evaluated from linear sweep voltammetry (LSV) experiments carried out on a rotating disk electrode (RDE) in O_2_-saturated 0.1 M KOH solutions at a scan rate of 10 mV s^−1^. The onset potential of Ni/Co-MOF nanoflakes for the ORR was at ~0.76 V. As shown in Fig. 4c, the corresponding K–L plots at various electrode potentials exhibit good linearity, indicating first-order reaction kinetics toward the ORR and a similar electron transmission number for the ORR at different potentials. According to the K–L equation, the transferred electron number in the O_2_ reduction process is calculated to be about 3.7 over the potential range ~0.05 to ~0.3 V; a four-electron process is the dominant pathway for the ORR at the Ni/Co-MOF nanoflake electrode. As a comparison, the ORR electrocatalytic activities of ZIF-67 and Ni-MOF nanoflakes were also investigated under the same conditions. Figure 4d shows the LSV curves of ZIF-67, Ni/Co-MOF nanoflakes, and Ni-MOF nanoflakes at a rotation rate of 1600 rpm. The highest onset potential and the largest cathodic current density were observed on the Ni/Co-MOF nanoflakes. As shown in Fig. S6a, b, ZIF-67 and Ni-MOF nanoflakes typically show a two-electron-dominated transfer pathway for the ORR at lower current densities, and this is proved by the lower average numbers of transferred electrons of 2.6 and 2.8 over the potential range ~0.05 to ~0.3 V, respectively, calculated from the slopes of K–L plots shown in Fig. S6c, d. Undoubtedly, Ni/Co-MOF nanoflakes possesses better electrocatalytic activity toward the ORR than Ni-MOF nanoflakes and ZIF-67 in the alkaline system. As confirmed from the Co 2*p* and Ni 2*p* XPS spectra, the dominant form of Co is Co^2+^, while that of Ni is Ni^3+^. Previous studies revealed that transition metals with mixed valences could provide donor–acceptor chemisorption sites for the reversible adsorption of oxygen and realize high electric conductivity for electron hopping between cations with different valences [46, 51], resulting in improved ORR electrocatalysis performance.

**Fig. 5:**
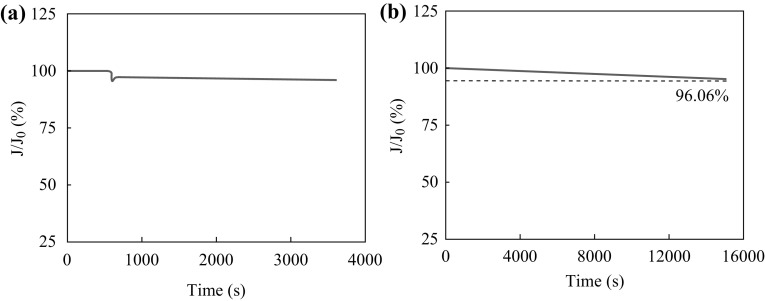
**a** Chronoamperometric responses of Ni/Co-MOF nanoflakes in O_2_-saturated 0.1 M KOH solution (1600 rpm) without and with 3 M methanol (added at 600 s). **b** Stability evaluation at −0.1 V and a rotation speed of 1600 rpm

The resistance to methanol crossover effects and durability are key factors for the practical application of the catalyst in fuel cells. As shown in Fig. 5a, the chronoamperometric responses of the Ni/Co-MOF nanoflakes upon addition of 3 M methanol were studied. With the addition of methanol, the superior stability of the Ni/Co-MOF nanoflakes for the ORR can be demonstrated from their negligible current decay, indicating superior tolerance against methanol crossover. In the catalytic activity durability test (Fig. 5b), Ni/Co-MOF nanoflakes exhibited a very low attenuation of 3.94% current loss at −0.1 V after 15,000 s, suggesting high stability of the Ni/Co-MOF nanoflakes. Therefore, the Ni/Co-MOF nanoflakes show promise for the ORR due to high catalytic activity, stability, and selectivity.

The results from the electrochemical performances for the supercapacitor and ORR studies indicate that the unique structure of the Ni/Co-MOF nanoflakes plays a role in optimizing the electrochemical performance of MOF materials. Significantly, the spherical microstructure assembled by interconnected MOF nanoflakes networks offers a continuous pathway for mass transportation, and the hollow nanocage formed in the textures may act as an ion-buffering reservoir for promoting transport of the electrolyte ion and oxygen, which bring more effective transmission (compared to the microporous only ZIF-67) leading to a high electrochemical performance for supercapacitors and the ORR. The synergistic effect of dual metallic (Ni^3+^/Ni^2+^ and Co^3+^/Co^2+^) ions in Ni/Co-MOF nanoflakes raised the reaction activity during the energy storage and conversion, thereby optimizing electron and charge transportation and accelerating the reaction kinetics (enhancing the activity). The enhanced conductivity of Ni/Co-MOF can be further demonstrated by the Nyquist plots. As shown in Fig. S5, Ni/Co-MOF exhibited a lower resistance (4.0 Ω) than ZIF-67 (8.5 Ω) and Ni-MOF (6.2 Ω), suggesting a higher conductivity of the Ni/Co-MOF that is probably attributed to electron hopping between cations with different valences [46, 51]. Moreover, the spherical microstructure of Ni/Co-MOF nanoflakes provides favorable layer-by-layer assembled nanoflakes, which effectively prevent the collapse of the nanoflakes (enhancing the stability). Additionally, to investigate the cation substitution effect, we replaced Ni^2+^ with Fe^2+^ ions in the structure; solid spheres were obtained instead of the nanosheet morphology, and they showed poor electrochemical performance (not shown here). Therefore, the choice of the Ni^3+^/Co^2+^ couple may be suitable for constructing a high-efficiency electrode. Accordingly, the Ni/Co-MOF nanoflakes exhibit better electrochemical performances for supercapacitors and the ORR than Ni-MOF nanoflakes and ZIF-67.

## Conclusions

We demonstrate an effective wet-chemical approach to achieve spherical hollow microstructures of assembled 2D Ni/Co-MOF nanoflakes and Ni-MOF nanoflakes. This approach leads to favorable Ni/Co-MOF nanoflake spherical microstructures with many exposed active sites. When applied in supercapacitors and the ORR, the Ni/Co-MOF exhibits remarkable performances with a specific capacitance of 530.4 F g^−1^ at 0.5 A g^−1^ (higher than that of Ni-MOF (306.8 F g^−1^) and ZIF-67 (168.3 F g^−1^)), good rate capability, and robust cycling performance with no capacity fading after 2000 cycles. Besides, Ni/Co-MOF nanoflakes can be used as an advanced non-noble metal catalyst for the ORR with its excellent oxygen reduction catalytic activity, good durability, and good methanol tolerance. Taking their robust electrochemical performance in supercapacitors and ORR into account, our work provides a new concept to design and synthesize rationally tunable structures of 2D MOF nanoflakes to improve the electrochemical performance for energy storage and conversion.

## Electronic supplementary material

Below is the link to the electronic supplementary material.

Supplementary material 1 (PDF 825 kb)
