# Supplementary material for: 2D MOF Nanoflake-Assembled Spherical Microstructures for Enhanced Supercapacitor and Electrocatalysis Performances
Source: Nanomicro Lett. 2017 Mar 28;9(4):43. doi: 10.1007/s40820-017-0144-6 (PMC6199045; doi:10.1007/s40820-017-0144-6)
Supplement: Supplementary file 1 — Supplementary material 1 (PDF 825 kb) [file 40820_2017_144_MOESM1_ESM.pdf]

Supporting Information for

2D MOF Nanoflake-Assembled Spherical-Micro-Structures for Enhanced Supercapacitor and Electrocatalysis Performances

Huicong Xia[1], Jianan Zhang[1,2,*], Zhao Yang[1], Shiyu Guo[1], Shihui Guo[3], Qun Xu[1,*]

[1]College of Materials Science and Engineering, Zhengzhou University, Zhengzhou 450001, People's Republic of China

[2]Key Laboratory of Advanced Energy Materials Chemistry (Ministry of Education), Nankai University, Tianjin 30071, People's Republic of China

[3]State Key Laboratory of Inorganic Synthesis and Preparative Chemistry, Jilin University, Changchun 130012, People's Republic of China

*Corresponding authors. E-mail: zjn@zzu.edu.cn, qunxu@zzu.edu.cn

Tel.: 86-15890185257

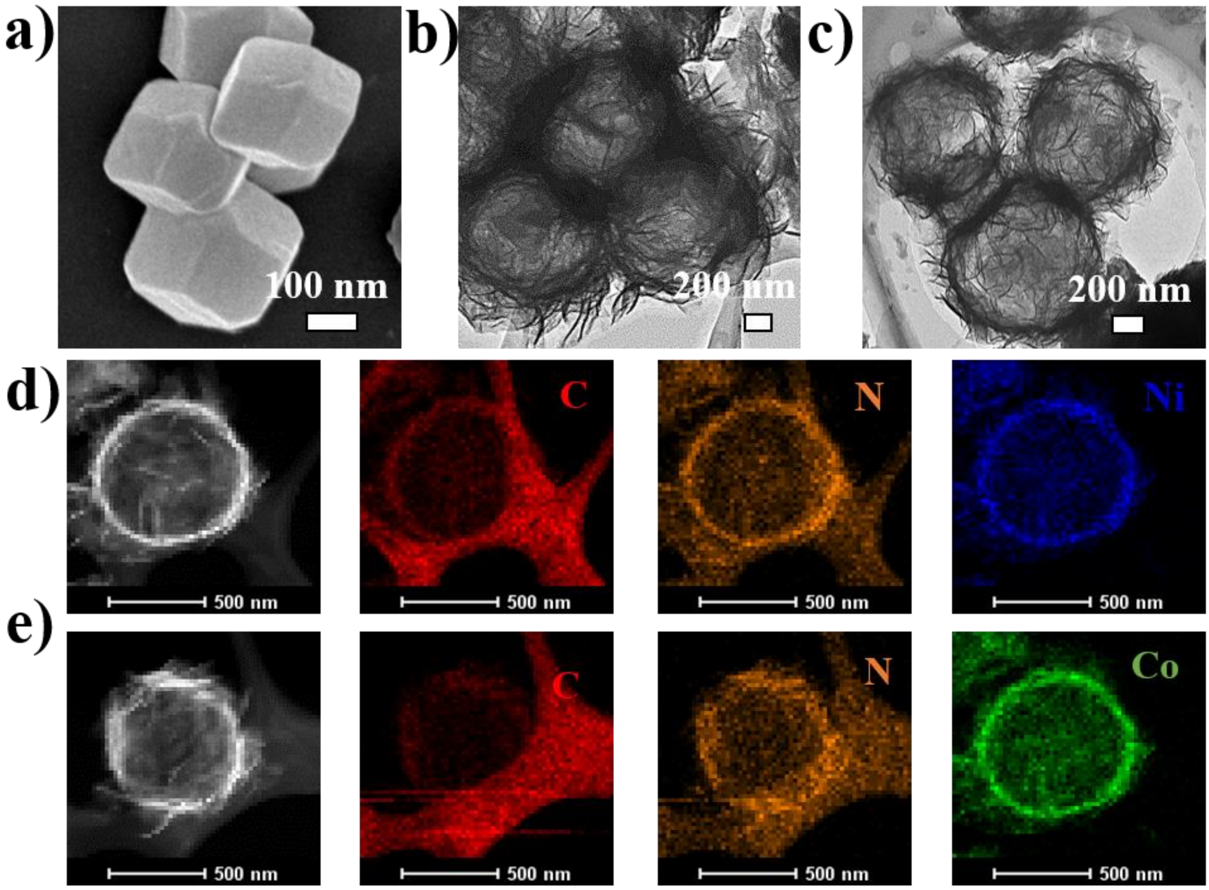

Fig. S1 **a** SEM images of ZIF-67. **b** TEM images of Ni/Co-MOF nanoflakes. **c** TEM images of Ni-MOF nanoflakes. **d** TEM-EDS mapping of Ni-MOF nanoflakes, and **e** TEM-EDS mapping of ZIF-67

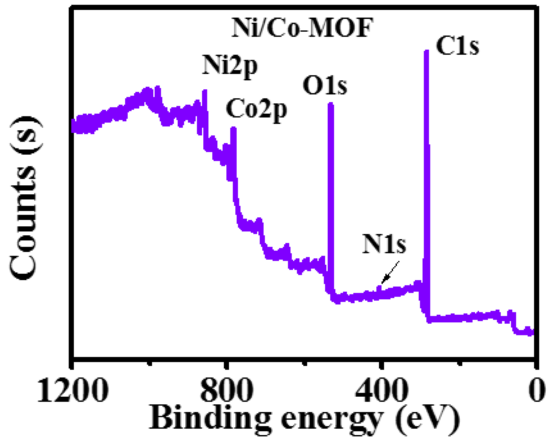

Fig. S2 XPS spectra of Ni/Co-MOF nanoflakes

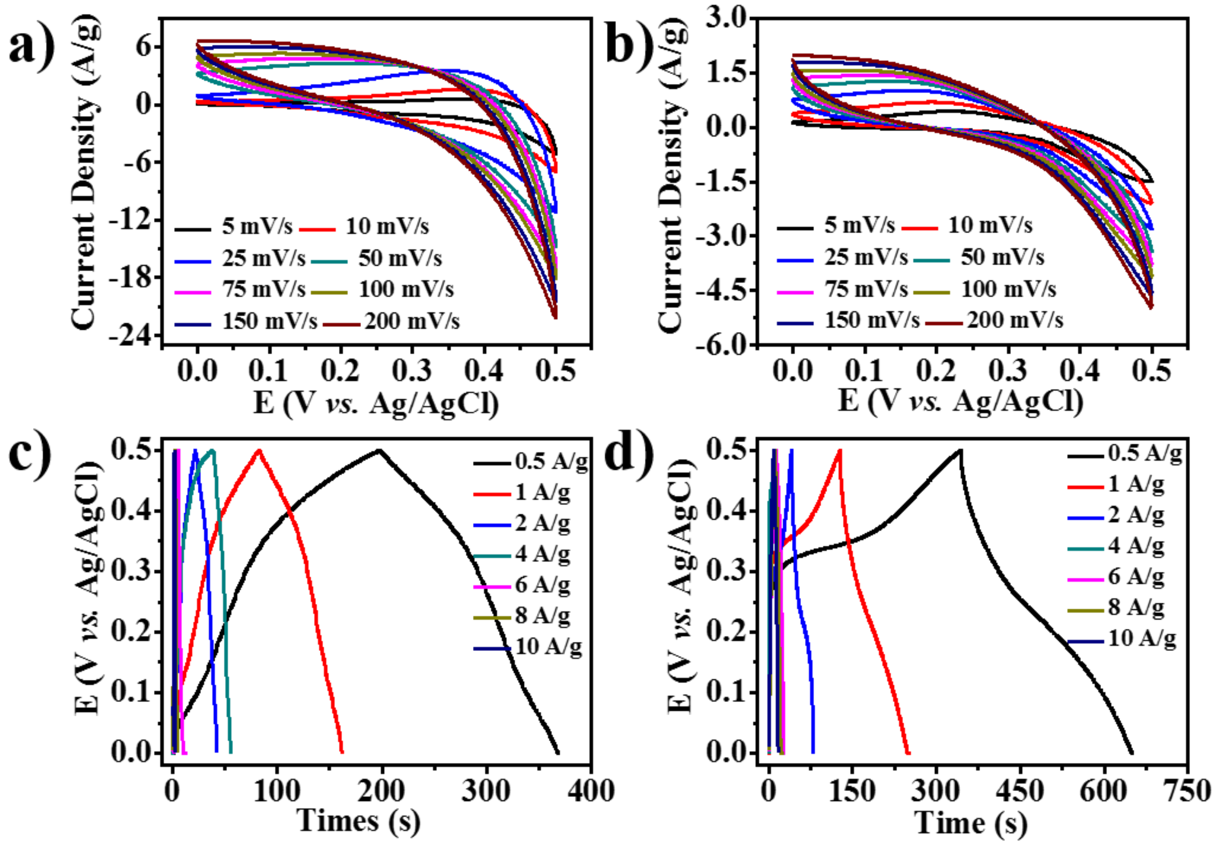

Fig. S3. **a** CV of as-prepared ZIF-67 eletrodes at different scan rates. **b** CV of as-prepared Ni-MOF nanoflakes eletrodes at different scan rates. **c** Galvanostatic charge/discharge curves of ZIF-67 at various current densities. **d** Galvanostatic charge/discharge curves of Ni-MOF nanoflakes at various current densities

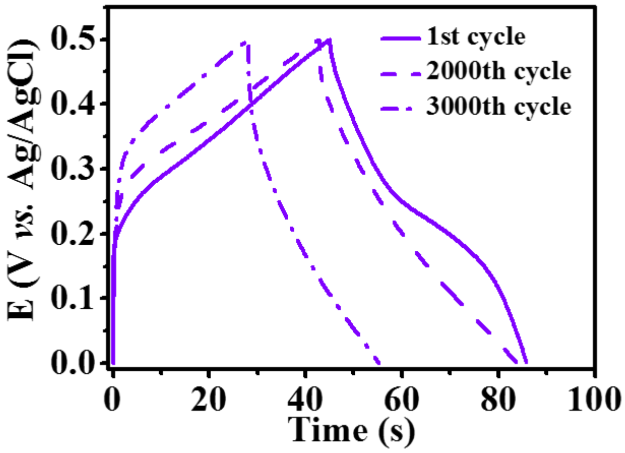

Fig. S4 Galvanostatic charge/discharge curves of Ni/Co-MOF nanoflakes supercapacitor before and after 2000 and 3000 cycles measured at 2 A g^{-1}

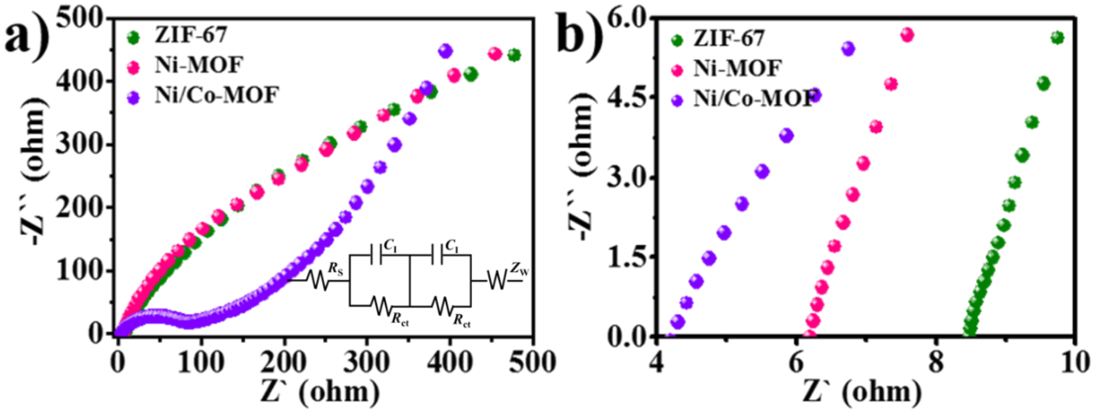

Fig. S5 Electrochemical impedance spectra measured in the frequency range of 10 mHz to 100 kHz at the open circuit voltage -0.1V with an alternate current amplitude of 5 mV. Inset: Equivalent circuit for the electrode-solution interface. C_I, double-layer capacitance. The Faradic impedance includes Z_w (the Warburg impedance) and R_{ct} (the charge-transfer resistance). R_s, spreading resistance

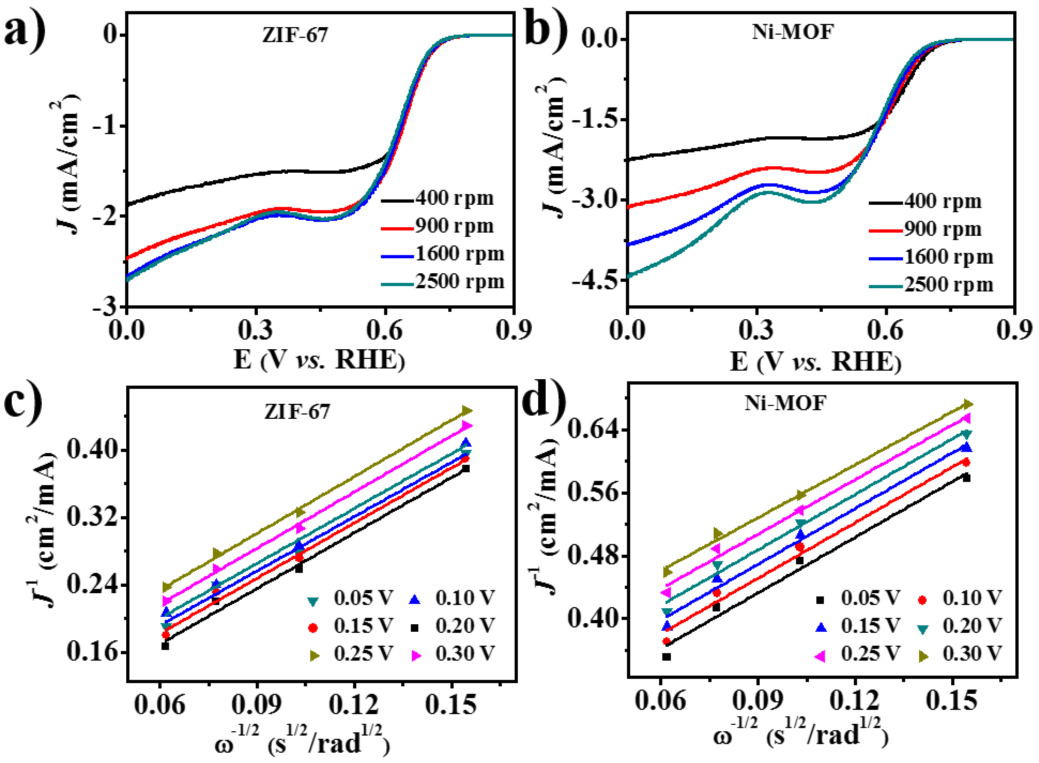

Fig. S6 a, b RDE polarization curves at different rotation speeds. Scan rate: 10 mV s^{-1}. **c, d** Koutech-Levich plots at various potentials

Table S1 Comparison of the capacities of Ni/Co-MOF with the recently reported MOF based materials

Sample	Electrolyte solution	Test Condition	Specific Capacitance (F g^{-1})	Resistance （ohm）	Ref.
Ni/Co-MOF	1.0 M LiOH	0.5 A g^{-1}	530.4	~4.0	This work
Co-MOF	1.0 M LiOH	0.5 A g^{-1}	230.5	-	23
N-doped Zn-MOF	6.0 M KOH	0.1 A g^{-1}	285.8	~6	48
ZIF-67	0.5 M H_2SO_4	20 mV s^{-1}	238	-	49
Co-MOF	6 M KOH	1 A g^{-1}	321	~5.23	50
